# Effects of Mulberry Fruit (*Morus alba* L.) Consumption on Health Outcomes: A Mini-Review

**DOI:** 10.3390/antiox7050069

**Published:** 2018-05-21

**Authors:** Hongxia Zhang, Zheng Feei Ma, Xiaoqin Luo, Xinli Li

**Affiliations:** 1 Department of Food Science, University of Otago, Dunedin 9016, New Zealand; zhanghongxia326@hotmail.com; 2 Department of Public Health, Xi’an Jiaotong-Liverpool University, Suzhou 215123, China; 3 School of Medical Sciences, Universiti Sains Malaysia, Kota Bharu 15200, Malaysia; 4 Department of Nutrition and Food Safety, School of Public Health, Xi’an Jiaotong University Health Science Center, Xi’an 710061, China; luoxiaoqin2012@mail.xjtu.edu.cn; 5 Department of Nutrition and Food Hygiene, School of Public Health, Medical College of Soochow University, Suzhou 215123, China; lixinli@suda.edu.cn

**Keywords:** mulberry, polyphenols, anthocyanins, health, nutrition

## Abstract

Mulberry (*Morus alba* L.) belongs to the Moraceae family and is widely planted in Asia. Mulberry fruits are generally consumed as fresh fruits, jams and juices. They contain considerable amounts of biologically active ingredients that might be associated with some potential pharmacological activities that are beneficial for health. Therefore, they have been traditionally used in traditional medicine. Studies have reported that the presence of bioactive components in mulberry fruits, including alkaloids and flavonoid, are associated with bioactivities such as antioxidant. One of the most important compounds in mulberry fruits is anthocyanins which are water-soluble bioactive ingredients of the polyphenol class. Studies have shown that mulberry fruits possess several potential pharmacological health benefits including anti-cholesterol, anti-obesity and hepatoprotective effects which might be associated with the presence of some of these bioactive compounds. However, human intervention studies on the pharmacological activities of mulberry fruits are limited. Therefore, future studies should explore the effect of mulberry fruit consumption on human health and elucidate the detailed compounds. This paper provides an overview of the pharmacological activities of mulberry fruits.

## 1. Introduction

Natural products have always been a rich source of biologically active compounds [1,2,3]. These substances present in fruits and vegetables have received increasing attention because of their antioxidant properties and potential strategy in reducing the risk of certain types of diseases such as metabolic syndrome [1,4,5]. About 50% of the drugs approved are natural products [5]. About 80% of the populations living in many countries rely on the phytomedicines and the plant-derived drug market is estimated to reach approximately $35 billion in 2020 [5,6].

Mulberry (*Morus alba* L.) belongs to the Morus genus of the Moraceae family [7]. Mulberry is also known as *Ramulus Mori* or Sangzhi [8]. To date, this genus has 24 species and 100 varieties that have been known [7]. Mulberry is a species native to China and has been widely cultivated in many regions including Asia, Africa, America, Europe and India [9]. China has planted mulberry for more than 5000 years and mulberry is a traditional Chinese edible fruit that can be eaten fresh [10]. According to traditional Chinese Medicine, mulberry fruits are used to improve eyesight and protect against liver damage [11]. They are grown to feed silkworms [12,13] The season of fresh mulberry fruit in China is usually less than 1 month. Mulberry fruits are difficult to preserve because they have high water content (i.e., ~80%) [11]. Mulberry has been used in traditional Oriental medicine to treat diabetes and premature white hair [14].

Mulberry fruits are appetising and low in calories [15]. Mulberry fruits have a sour taste with a pH < 3.5, providing a more concentrated flavour for fruit production and fresh-eating [16]. Mulberry fruits possess several potential pharmacological properties including anti-cholesterol, anti-diabetic, antioxidative and anti-obesity effects [8,17,18,19]. These pharmacological properties are due to the presence of polyphenol compounds including anthocyanins, however, different colours of mulberry fruits even from the same species may have different amounts of anthocyanins [20]. Cyanidin-3-rutinoside and cyanidin-3-glucoside are the major anthocyanins isolated from mulberry fruits [21,22].

Although different mulberry varieties with the same genotype are likely to have differences in nutritional values and pharmacological properties [23], the aim of this work was to review some potential roles of mulberry fruits (*Morus alba* L.) and their bioactive compounds in health. Also, some of the potential mechanisms of their actions will be discussed briefly. We hope that this work would provide a valuable reference resource for future studies in this area.

### Search Strategy

An electronic literature search was conducted using Google Scholar, Medline (OvidSP) and PubMed until February 2018. Additional articles were identified and obtained from references in the retrieved articles. Search terms included combinations of the following: mulberry, fruits, hypertension, diabetes, anti-tumour, hepatoprotective, anti-obesity, anti-oxidative stress and phytochemicals. For the purpose of this mini-review, the search was restricted to experimental, epidemiological and clinical studies published in English that address the phytochemical constituents and pharmacological properties of mulberry fruits (*Morus alba* L.).

## 2. Phytochemical Compounds

**Figure 1:**
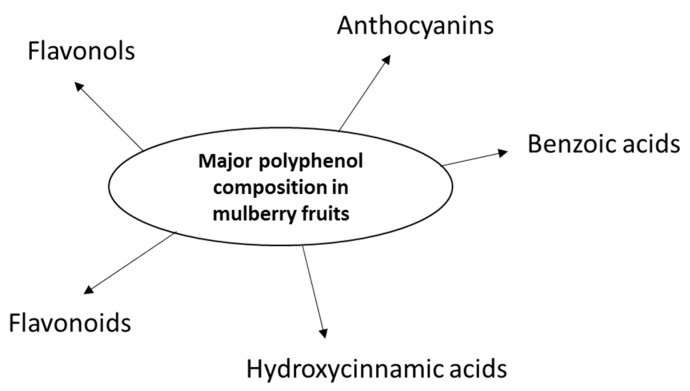
Major polyphenol composition in mulberry fruits.

Compared with mulberry leaves and barks, mulberry fruits are less commonly used in traditional Chinese Medicine. The possible reasons might be due to the lack of awareness of their health benefits and limited production [24]. However, there is increasing interest in isolating and quantifying the phytochemical compounds from mulberry fruits. This is because mulberry fruits can be also consumed as foods [24]. Mulberry fruits have strong antioxidant property which is due primarily to the presence of polyphenols [25]. Figure 1 shows the major polyphenol composition found in mulberry fruits.

Phytochemical compounds of mulberry fruits (*Morus nigra*, *Morus indica* and *Morus rubra*) have been reported in several studies [26,27,28]. Kang, Hur, Kim, Ryu and Kim [17] isolated cyanidin-3-*O*-β-d-glucopyranoside (C3G) from 1% HCI-MeOH mulberry fruit extracts using Amberlite IRC-50 ion exchange chromatography. C3G was identified and quantified by liquid chromatography-mass spectroscopy (LC-MS) and High-Performance Liquid Chromatography (HPLC) [17]. C3G is an aglycon of anthocyanin that has inflammation-suppressing and free radical scavenging activity, which might protect against endothelial dysfunction [17].

In a study assessing the polyphenolic composition of five major mulberry fruit varieties (i.e., Pachungsipyung, Whazosipmunja, Suwonnosang, Jasan and Mocksang) cultivated in Korea using spectrophotometric methods, Bae and Suh [29] reported that the total phenols, total anthocyanins, coloured (ionised) anthocyanins and total flavanols ranged from 960 to 2570 µg/g gallic acid equivalents, 137 to 2057 µg/g malvidin-3-glucoside equivalents, 10 to 190 µg/g malvidin-3-glucoside equivalents and 6 to 65 µg/g catechin equivalents.

Kusano, Orihara, Tsukamoto, Shibano, Coskun, Guvenc and Erdurak [24] isolated five new nortropane alkaloids (i.e., 2α,3β-dihydroxynortropane, 2β,3β-dihydroxynortropane, 2α,3β-6exo-trihydroxynortropane, 2α,3β,4α-trihydroxynortropane, 3β,6exo-dihydroxynortropane) along with nor-ψ-tropine from ripened mulberry fruits grown in Turkey. In addition, Kusano, Orihara, Tsukamoto, Shibano, Coskun, Guvenc and Erdurak [24] also isolated and determined the new structures of six amino acids, which were morusimic acid A, morusimic acid B, morusimic acid C, morusimic acid D, morusimic acid E and morusimic acid F using spectroscopic data.

Kim, et al. [30] identified five pyrrole alkaloids in mulberry fruits, which were morrole B, morrole C, morrole D, morrole E and morrole F based on spectroscopic data. In addition, the authors [30] also isolated 11 pyrrole alkaloids, which were 4-[formyl-5-(hydroxymethyl)-1H-pyrrol-1-yl]butanoate, 2-(5-hydroxymethyl-2′,5′-dioxo-2′,3′,4′,5′-tetrahydro-1′H-1,3′-bipyrrole)carbaldehyde, 4-[formyl-5-(hydroxymethyl)-1H-pyrrol-1-yl]butanoate, 4-[formyl-5-(methoxymethyl)-1H-pyrrol-1-yl]butanoic acid, methyl 2-[2-formyl-5-(methoxymethyl)-1H-pyrrole-1-yl]propanoate, 2-(5′-hydroxymethyl-2′-formylpyrrol-1′-yl)-3-phenyl-propionic acid lactone, methyl 2-[2-formyl-5-(methoxymethyl)-1H-pyrrol-1-yl]-3-(4-hydroxyphenyl)propanoate, 2-(5′-hydroxymethyl-2′-formylpyrrol-1′-yl)-3-(4-hydroxyphenyl)-propionic acid lactone, 2-(5-hydroxymethyl-2-formylpyrrole-1-yl)propionic acid lactone, 2-(5-hydroxymethyl-2-formylpyrrol-1-yl)isovaleric acid lactone, 2-(5-hydroxymethyl-2-formylpyrrole-1-yl)isocaproic acid lactone and 2-[2-formyl-5-(hydroxymethyl)-1-pyrrolyl-]3-methylpentanoic acid lactone.

Natić, et al. [31] isolated epigallocatechin, epigallocatechin gallate, gallocatechin, gallocatechin gallate, isorhamnetin glucuronide, isorhamnetin hexoside, isorhamnetin hexosylhexoside, kaempferol glucuronide, kaempferol hexoside, kaempferol hexosylhexoside, kaempferol rhamnosylhexoside, morin and naringin from mulberry fruits grown in Vojvodina, North Serbia. Quercetin glucoronide, quercetin hexoside, quercetin hexosylhexoside, quercetrin from mulberry fruits were also isolated using ultra HPLC (UHPLC) system coupled to a high resolution mass spectrophotometer [31]. In addition, the authors [31] also reported the presence of cyanidin galloylhexoside, cyanidin hexoside, cyanidin hexosylhexoside, cyanidin pentoside, cyanidin rhamnosylhexoside, delphinidin acetylhexoside, delphinidin hexoside, delphinidin rhamnosylhexoside, pelargonidin hexoside, pelargonidin rhamnosylhexoside and petunidin rhamnosylhexoside from mulberry fruits.

Qin, et al. [32] isolated cyanidin 3-*O*-glucoside, cyanidin 3-*O*-rutinoside, pelargonidin 3-*O*-glucoside and pelargonidin 3-*O*-rutinoside ultraviolet-visible from mulberry fruits grown in Shaanxi, China using UV-Visible spectroscopy, HPLC-pulsed amperometric detector (PAD), LC-MS and proton nuclear magnetic resonance (1HNMR). Du, et al. [33] isolated cyanidin 3-*O*-β-d-galactopyranoside, cyanidin 3-*O*-β-d-glucopyranoside and cyanidin 7-*O*-β-d-glucopyranoside from mulberry fruits bought from local stores in Hangzhou, China. In addition, the authors [33] also isolated cyanidin 3-*O*-(6′′-*O*-α-rhamnopyranosyl-β-d-galactopyranoside) and cyanidin 3-*O*-(6′′-*O*-α-rhamnopyranosyl-β-d-glucopyranoside) from mulberry fruits. While Memon, et al. [34] isolated gallic acid, protocatechuic acid, protocatechuic aldehyde, *p*-hydroxybenzoic acid, vanillic acid, chlorogenic acid, syringic acid, syringealdehyde and m-coumaric acid from mulberry fruits grown in Pakistan. A study by Peng, et al. [35] identified eight major compounds which were gallic acid, chlorogenic acid, protocatechuic acid, rutin, caffeic acid, 3-caffeoyl quinic acid, 4-caffeoyl quinic acid and quercetin-3-*O*-glucoside in mulberry fruit water extract.

Another study by Kim, et al. [36] identified four pyrrole alkaloids from mulberry fruits planted in Chonbuk, Korea which were 2-formyl-5-(hydroxymethyl)-1H-pyrrole-1-butanoic acid, 5-(hydroxymethyl)-1H-pyrrole-2-carboxaldehyde, 2-formyl-1H-pyrrole-1-butanoic acid and 2-formyl-5-(methoxymethyl)-1H-pyrrole-1-butanoic acid. In addition, the authors [36] also isolated a new pyrrole alkaloid, which was morrole A. All the structures of isolated pyrrole alkaloids were determined using 1D and 2D nuclear magnetic resonance (NMR) analyses [36].

Isabelle, et al. [37] reported the presence of 3-caffeoyl quinic acid, 5-caffeoyl quinic acid, cyanidin-3-glucoside, 4-caffeoyl quinic acid, cyanidin-3-rutinoside, pelargonidin-3-glucoside, rutin, quercetin and kaempferol-3-rutinoside in the Chinese mulberry fruit cultivar Guo-2. In addition, the authors [37] also found the presence of α-tocopherol, α-tocotrienol, δ-tocopherol, γ-tocopherol, β-carotene, lutein, neoxanthin and violaxanthin in the Chinese mulberry fruit cultivar Bei-2-5, Guiyou-154, Heipisang, Xuan-27 and Tang-10. Rutin, 1-deoxynojirimycin (DJN), cyanidin-3-*O*-β-glucoside, cyanidin-3-*O*-β-rutinoside, resveratrol and oxyresveratrol were also present in the Chinese mulberry fruits [38,39].

Wang, Xiang, Wang, Tang and He [15] isolated quercetin-3-*O*-β-d-glucopyranoside, quercetin 3-*O*-(6′′-*O*-acetyl)-β-d-glucopyranoside, quercetin 3-*O*-β-d-rutinoside, quercetin 7-*O*-β-d-glucopyranoside, quercetin 3,7-di-*O*-β-d-glucopyranoside, kaempferol 3-*O*-β-d-glucopyranoside, kaempferol 3-*O*-β-d-rutinoside, isobavachalcone, 2,4,2′,4′,-tetrahydroxy-3′-(3-methyl-2-butenyl)-chalcone (morachalcone), (2E)-1-[2,3-dihydro-4-hydroxy-2-(1-methylethenyl)-5-benzofuranyl]-3-(4-hydroxyphenyl)-1-propanone, 5,7,3′-trihydroxy-flavanone-49-*O*-β-d-glucopyranoside, 5,7,4′-trihydroxy-flavanone-3′-*O*-β-d-glucopyranoside, dihydrokaempferol 7-*O*-ß-d-glucopyranoside, 2-*O*-(3,4-dihydroxybenzoyl)-2,4,6-trihydroxyphenylacetic acid, 2-*O*-(3,4-dihydroxybenzoyl)-2,4,6-trihydroxyphenylmethylacetate (jaboticabin), *p*-hydroxybenzoic acid, protocatechuic acid, 3-methoxy-4-hydroxybenzoic acid (vanillic acid), protocatechuic acid methyl ester, protocatechuic acid ethyl ester, 4-hydroxyphenylacetic acid methyl ester, 5,7-dihydroxychromone, 2-(4-hydroxyphenyl)ethanol (tyrosol) and pyrocatecholin in ethyl acetate-soluble extract of mulberry fruits. The authors [15] determined the structures of isolated compounds based on MS and NMR analysis.

Jiang and Nie [40] reported that mulberry fruit cultivar Hetianbaisang contains many types of essential amino acids (i.e., isoleucine, leucine, threonine, lysine, valine, phenylalanine, tyrosine, tryptophan, histidine, methionine and cysteine) and seven non-essential amino acids (i.e., arginine, alanine, proline, glutamic acid, glycine, serine and aspartic acid). In addition, the authors [40] also found the presence of minerals including potassium, calcium, magnesium, iron, sodium, zinc, copper, selenium and manganese in mulberry fruit cultivar Hetianbaisang. Mulberry fruit cultivar Hetianbaisang also contains organic acids including malic acid, succinic acid, citric acid, tartaric acid, acetic acid [40]. In addition, linoleic acid, myristic acid, stearic acid, palmitic acid and α-linoleic acid were also detected in mulberry fruit cultivar Hetianbaisang [40].

Yang, Yang and Zheng [11] reported that the total phenolics, total flavonoids and anthocyanins in the freeze-dried powder of mulberry fruits were 23.0 mg/g gallic acid equivalents, 3.9 mg/g rutin equivalents, 0.87 mg/g cyanidin-3-glucoside equivalents, respectively. The major flavonol in mulberry fruit powder was rutin (0.43 mg/g), followed by morin (0.16 mg/g), quercetin (0.01 mg/g) and myricetin (0.01 mg/g) [11]. HPLC was used to determine the flavonols in mulberry fruit powder [11]. In addition, the freeze-dried powder of mulberry fruits also contained 1.20 mg/g ascorbic acid, 0.32 mg/g vitamin E and 243.0 mg/g dietary fibre [11].

Fatty acid content and composition of mulberry can vary according to different ecological conditions. For example, Yang, Yang and Zheng [11] found that Chinese mulberry fruits had 7.55% total lipids, with 87.5% of unsaturated fatty acids. The highest fatty acid content in Chinese mulberry fruits was linoleic acid C18:2 (79.4%), followed by palmitic acid C16:2 (8.6%) and oleic acid C18:1 (7.5%) [11]. In addition, Chinese mulberry also contained 0.6% α-linolenic acid C18:3 [11]. Although the highest fatty acid content in Turkish mulberry was linoleic acid C18:2 (57.3%) followed by palmitic acid C16:0 (22.4%); no presence of linolenic acid C18:3 was reported [7].

Different colours of mulberry fruits (*M. alba* L) such as red, purple and purple-red have been reported [41]. Aramwit, Bang and Srichana [41] reported that purple mulberry fruit extract had higher contents of total sugars and anthocyanins than red and purple-red mulberry fruit extracts. This is because sugars are needed as the precursors to synthesis anthocyanins [41]. However, red mulberry fruit had a higher ascorbic acid and ß-carotene than purple and purple-red mulberry fruit extracts [41].

Many volatile compounds have also been found in mulberry fruits [42]. Calin-Sanchez, Martinez-Nicolas, Munera-Picazo, Carbonell-Barrachina, Legua and Hernandez [42] reported that volatile compounds found in mulberry fruits grown in Spain included acetic acid, 3-hydroxyl-2-butanone, ethyl butyrate, ethyl acetate, 3-methylbutanal, 2-methybutanal, heptanal, methional, hexanal, trans-2-hexanal, 2-octenone,hexanoic acid, benzaldehyde, methyl hexanoate, 2-ethylhexanal, octanal, limonene, 6-methyl-5-hepten-2on, ethyl hexanoate, 2,4-nonanadienal, phenylacetaldehyde, trans-2-octenal, cis-α-ocimene, terpinonene, 2-nonanone, nonanal, octanoic acid, cis-2-nonenal, dodecanoic acid, terpinen-4-ol, ethyl octanoate, ethyl dodecanoate, decanal, decanoic acid and ethyl decanoate. The authors [42] suggested that these volatile compounds in mulberry fruits might present better sensory profiles for the market demands from consumers.

Chen, et al. [43] reported that the levels of phenolic compounds in mulberry fruits are higher than blackberry, blueberry, raspberry and strawberry, suggesting that mulberry fruits can be used as good sources of phenolic compounds. Therefore, mulberry fruits are rich in diverse phenolic compounds including polyhenols, anthocyanins and flavonoids.

## 3. Pharmacological Properties

As mentioned previously, mulberry fruits are rich in anthocyanins [44], which have attracted attention of researchers and consumers because of their potential pharmacological activities on health [45,46,47,48,49]. Anthocyanins from mulberry fruits can inhibit the oxidation of low-density lipoprotein (LDL) and scavenge free radicals [33,50]. Many studies have showed that mulberry leaves exhibit a wide range of pharmacological activities [51,52,53,54,55,56,57]. However, there are limited studies that have been conducted on the pharmacological properties of mulberry fruits [15,58,59]. Also, most studies have been conducted in animal models using mulberry fruits as a dietary supplement [15,58,59]. Although existing literature shows that there is relationship between mulberry fruit consumption and improved health outcomes, these studies often infer a causal correlation between a bioactive substance of mulberry fruits and the observed health outcomes. This approach is more likely to oversimplify the complicated body mechanisms that will eventually lead to the observed health outcomes. Therefore, the conclusions based on such studies should always be interpreted with caution [60] because the observed health outcomes may not be attributed to the action of a single bioactive compound of mulberry fruits.

### 3.1. Hypolipidemic

Cardiovascular disease (CVD) is one of the most common causes of deaths, with about 17 million people die of CVD (including stroke and coronary heart disease) every year worldwide [61,62]. It is estimated that CVD will continue to be the largest contributor to global mortality in the future [63] Hyperlipidemia is one of the major risk factors for CVD [64]. Therefore, an increasing focus has been reported in research studies that determine the effectiveness of natural alternative medicine in reducing blood lipid levels [11]. This is because majority of the hypolipidemic drugs can potentially cause side effects and they are expensive [11].

Yang, Yang and Zheng [11] reported that rat fed with high fat diet supplemented with 5% or 10% mulberry fruit powder had a significant decrease in the concentration of serum and liver triglyceride, total cholesterol and serum LDL cholesterol. An increase in the serum high-density lipoprotein (HDL) cholesterol was reported in rat fed with high fat diet supplemented with 5% or 10% mulberry fruit powder [11]. It is suggested that the presence of dietary fiber in mulberry fruits inhibits the hepatic lipogenesis and increases LDL-receptor activity [65]. In addition, the authors suggested that mulberry fruits might have a hypolipidemic effect because mulberry fruits have high content of dietary fiber and linoleic acid [11].

Chen, Liu, Hsu, Huang, Yang and Wang [50] reported that New Zealand white rabbits fed with high cholesterol diet (HCD) (containing 95.7% standard Purina chow, 3% lard oil and 1.3% cholesterol) plus 0.5% or 1.0% water extract of mulberry fruits for 10 weeks had lower levels of total cholesterol, LDL cholesterol, and triglycerides than those fed with only lard oil diet. The authors [50] also showed that rabbits fed with HCD plus 0.5% or 1.0% water extract of mulberry fruits had significantly reduced severe atherosclerosis in the aorta by 42–63% and these findings were supported by histopathological examination of blood vessel of rabbits. The effect of water extract of mulberry fruits on the levels of total cholesterol and LDL cholesterol was reported to be dose-dependent [50]. No adverse effects on the changes of liver or renal functions in rabbits fed with HCD plus 0.5% or 1.0% water extract of mulberry fruits were reported [50].

In a randomised controlled study of 58 hypercholesterolemic adults aged 30–60 years, Sirikanchanarod, et al. [66] reported that after 6 weeks of 45 g freeze-dried mulberry fruit consumption (325 mg anthocyanins), the intervention group had a significantly lower level of total cholesterol and LDL (both *p*-values < 0.001) than the control group. The authors [66] suggested that mulberry fruits might be used as an alternative treatment for hypercholesterolemic patients. Therefore, the consumption of mulberry fruits might reduce the risk of atherosclerosis because mulberry fruits possess anti-hyperlipidemic and anti-oxidative abilities to prevent the oxidation of LDL [50].

### 3.2. Anti-Diabetic

Diabetes is characterised by hyperglycemia which results from the defects of secretion of insulin [67]. It is associated with a series of health complications including CVD and failure of various organs [67]. Jiao, Wang, Jiang, Kong, Wang and Yan [59] reported that diabetic rats fed with two different fractions of mulberry fruit polysaccharides (MFP50 and MFP90) for seven weeks had a significant decrease in the levels of fasting glucose, fasting serum insulin, homeostasis model of assessment-insulin resistance, triglyceride and oral glucose tolerance test-area under the curve. The MFP50 and MFP90 had a final ethanol concentration of 50% and 90%, respectively [59]. When compared with diabetic rats fed with pure water, diabetic rats fed with MFP50 and MFP90 had a lower serum insulin level at a rate of 26.5% and 32.5%, respectively [59]. The MFP50 group had a significant increase in the level of HDL cholesterol and the proportion of HDL cholesterol to total cholesterol [59]. The authors [59] also found that both MFP50 and MFP90 reduced the levels of serum alanine transaminase (ALT), suggesting that they have potential hepatoprotective effects. Although MFP50 had a more stable hypoglycemic effect than MFP90, MFP90 had a better hypolipidemic effect than MFP50 [59].

Similar findings were also reported by Guo, Li, Zheng, Xu, Liang and He [58] who found that diabetic rats fed with mulberry fruit polysaccharides for 2 weeks had a decrease in fasting blood glucose. Another study by Wang, Xiang, Wang, Tang and He [15] reported that diabetic rats fed with ethyl acetate-soluble extract of mulberry fruits for 2 weeks had a significant decrease in the levels of fasting blood glucose and glycosylated serum protein. The authors [15] also found that ethyl acetate-soluble extract of mulberry fruits had significantly increased the antioxidant activities of catalase (CAT), glutathione peroxidase (GSH-Px) and superoxide dismutase (SOD) in diabetic rats. Ethyl acetate-soluble extract of mulberry fruits also possesses strong α-glucoside inhibitory activity and radical-scavenging activities against 2,2-diphenyl-1-picrylhdrazyl (DPPH) and superoxide anion radicals [15]. A study by Xu, et al. [68] reported that diabetic mice fed with mulberry fruit polysaccharides had a lower level of haemoglobin A1c (HbA1c) and a reduction in streptozotocin (STZ)-lesioned pancreatic cells. In addition, diabetic mice fed with mulberry fruit polysaccharides also had an increase in insulin level and B-cell lymphoma 2 (bcl-2) expression [68].

Yan, et al. [69] reported that male C57BL6/J genetic background (db/db) mice fed with anthocyanin extract of mulberry fruit in the doses of 50 and 125 mg/kg body weight per day for 8 weeks had a significant decrease in the levels of cholesterol, fasting blood glucose, leptin, serum insulin and triglyceride as well as an increase in adiponectin level. Therefore, the authors [69] suggested that anthocyanin extract of mulberry fruit can be used to improve the resistance of insulin and leptin. Taken together, these results [15,58,59] suggest that mulberry fruits might play an important role in the treatment of diabetes because of their anti-hyperglycemic and anti-hyperlipidemic effects.

### 3.3. Anti-Obesity

Several studies have shown that obesity plays a major role in contributing to dyslipidemia [70,71,72,73]. Lim, et al. [74] reported that high fat diet-induced obese mice fed with a combination of mulberry leaf extract and mulberry fruit extract at low and high doses had a significant decrease in body weight gain, fasting plasma glucose, insulin and homeostasis model assessment of insulin resistance. The low dose of combination of mulberry leaf extract and mulberry fruit extract was 133 mg mulberry leaf extract and 67 mg mulberry fruit extract/kg/day, while the high dose of combination of mulberry leaf extract and mulberry fruit extract was 333 mg mulberry leaf extract and 167 mg mulberry fruit extract/kg/day [74]. The high dose of combination of mulberry leaf extract and mulberry fruit extract had significantly improved the glucose control [74]. In addition, the high dose of combination of mulberry leaf extract and mulberry fruit extract also decreased the protein levels of manganese superoxide dismutase, inducible nitric oxide synthase, monocyte chemoattractant protein-1, C-reactive protein (CRP), tumour necrosis factor-α and interleukin-1 [74]. Therefore, it is suggested that the combination of mulberry leaf extract and mulberry fruit extract possess the anti-obesity and anti-diabetic properties by modulating oxidative stress and inflammation induced by obesity [74].

Peng, Liu, Chuang, Chyau, Huang and Wang [35] reported that male hamsters fed with mulberry fruit water extract for 12 weeks had a lower high fat diet-induced body weight and visceral fat, accompanied with a decrease in serum triacylglycerol, cholesterol, LDL/HDL ratio and free fatty acid. In addition, mulberry fruit water extract also reduced fatty acid synthase and 3-hydroxy-3-methylglutaryl-coenzyme A (HMG-CoA) reductase and elevated hepatic peroxisome proliferator-activated receptor α and carnitine palmitoyltransferase-1 [35]. No physiological burdens in terms of levels of serum blood urea nitrogen, creatinine, potassium and sodium ions were exerted by the administration of mulberry fruit extract [35]. The authors [35] suggested that mulberry fruit water extracts regulate lipolysis and lipogenesis, which can be used to reduce the body weight.

### 3.4. Anti-Tumour

Gastrointestinal tract cancers are also one of the most common types of cancers in the world [75,76] and *Helicobacter pylori* is one of the common suspects in triggering the gastric carcinogenesis [77,78]. Huang, et al. [79] reported that after male balb/c nude mice were fed with anthocyanin-rich mulberry fruit extract for 7 weeks, atypical glandular cells (AGS) tumour xenograft growth in mice was inhibited, suggesting that anthocyanins from mulberry fruits might be used to prevent gastric carcinoma formation.

### 3.5. Hepatoprotective

In a study investigating the protective effect of mulberry fruit marc (the solid component after juicing) anthocyanins on carbon tetrachloride (CC14)-induced liver fibrosis in male Sprague Dawley rats, Li, et al. [80] reported that rats fed with mulberry fruit marc anthocyanins had a decrease in the levels of ALT, aspartate amino transferase, collagen type-III hyaluronidase acid and hydroxyproline. Another study by Chang, et al. [81] reported that mulberry fruit extracts suppressed the synthesis and enhanced the oxidation of fatty acids. Therefore, the mulberry fruits might prevent the non-alcoholic fatty liver disease.

### 3.6. Protective against Cytotoxicity and Oxidative Stress

In a study investigating the protective effect of mulberry fruit extract against ethyl carbamate (EC)-induced cytotoxicity in human liver HepG2 cells, Chen, Li, Bao and Gowd [43] reported no decrease in cell viability with the treatments of mulberry fruit extract (0.5 mg/mL, 1.0 mg/mL and 2.0 mg/mL). Therefore, the authors [43] suggested that mulberry fruits can be used to protect against EC-induced cytotoxicity and oxidative stress. Also, in a study investigating the effect of mulberry fruit consumption on the anti-fatigue activity in mice using a weight-loaded swimming test, Jiang, Guo, Xu, Huang, Yuan and Lv [44] reported that mice fed with mulberry juice purification and mulberry marc purification had an increase endurance capacity than the control group. The authors [44] suggested that the presence of anthocyanins in mulberry fruits might act as an antioxidant to reduce exercise-induced oxidative stress and physical fatigue.

### 3.7. Protective against Brain Damage

Kang, Hur, Kim, Ryu and Kim [17] reported that that C3G isolated from mulberry fruit extracts had shown a cytoprotective effect on PC12 cells exposed to hydrogen peroxide in vitro and a neuroprotective effect on cerebral ischemic damage caused by oxygen glucose deprivation (OGD) in vivo. Therefore, it is suggested that mulberry fruits possess neuroprotective effects in vivo and vitro ischemic oxidative stress [17]. Table 1 shows an overview of animal studies investigating the pharmacological properties of mulberry fruits.

### 3.8. Adverse Effects

Due to a limited number of human studies, it is difficult to assess the safety of mulberry fruit consumption. Moreover, there is insufficient evidence regarding the recommended consumption of mulberry fruits (i.e., dosage) and its treatment duration. It is necessary that all future clinical studies that investigate the effects of mulberry fruit consumption on health should follow the Consolidated Standards of Reporting Trials (CONSORT) guidelines for generating scientifically rigorous evidence [82,83,84].

## 4. Conclusions and Future Research

Literature reviews have highlighted that mulberry fruits contain high content of polyphenolic compounds and antioxidants [85]. This suggests that there are many opportunities for the food and healthcare industry to explore the health benefits of mulberry fruits because there is a potential growing market for mulberry fruits. However, the contents of bioactive compounds such as anthocyanins, alkaloids, flavonoids and polyphenols are dependent on the cultivars. Although the bioactive compounds may work synergistically to promote health, such claims still require further investigation in order to establish the causative relationship between mulberry fruit consumption and health.

There are limited studies with sufficient data to support whether mulberry fruits are beneficial to human health especially in terms of the management and prevention of chronic diseases such as diabetes and CVD. The majority of the studies that reported beneficial effects of mulberry fruits on health are animal-based studies. Moreover, these studies used different varieties of mulberry fruits, types of solvents and methods of preparation, which cause the evaluation of activity of mulberry fruits to be difficult and these studies involve quite heterogeneous data. Therefore, larger well-designed, randomised controlled trials are needed to examine the effects of mulberry fruit consumption on human health. Similar to other plants and food products [1,86], the fate of polyphenol compounds in the body, especially after undergoing intestinal transformations by enzymes produced by gut microbiota should also be addressed. The elucidation of some active ingredient structures in mulberry fruits and their mechanisms in promoting pharmacological properties are also worthy of further research.

## Figures and Tables

**Table 1:** An overview of animal studies investigating the pharmacological properties of mulberry fruits.

Pharmacological Properties	References
Hypolipidemic	Yang et al. [11]; Chen et al. [50]; Sirikanchanarod et al. [66]
Anti-diabetic	Wang et al. [15]; Jiao et al. [59]; Guo et al. [58]; Xu et al. [68]; Yan et al. [69]
Anti-obesity	Peng et al. [35]; Lim et al. [74]
Anti-tumour	Huang et al. [79]
Hepatoprotective	Li et al. [80]; Chang et al. [81]
Protective against cytotoxicity and oxidative stress	Jiang et al. [44]; Chang et al. [81]
Protective against brain damage	Kang et al.[17]
